# Catalytic Co‐Conversion of CH_4_ and CO_2_ Mediated by Rhodium–Titanium Oxide Anions RhTiO_2_^−^

**DOI:** 10.1002/anie.202103808

**Published:** 2021-05-17

**Authors:** Yuan Yang, Ya‐Ke Li, Yan‐Xia Zhao, Gong‐Ping Wei, Yi Ren, Knut R. Asmis, Sheng‐Gui He

**Affiliations:** ^1^ State Key Laboratory for Structural Chemistry of Unstable and Stable Species Institute of Chemistry Chinese Academy of Sciences Beijing 100190 P.R. China; ^2^ Wilhelm-Ostwald Institut für Physikalische und Theoretische Chemie Universität Leipzig Linnéstrasse 2 04103 Leipzig Germany; ^3^ University of Chinese Academy of Sciences Beijing 100049 P.R. China; ^4^ Beijing National Laboratory for Molecular Sciences and CAS Research/Education Centre of Excellence in Molecular Sciences Beijing 100190 P.R. China; ^5^ Fritz-Haber-Institut der Max-Planck-Gesellschaft Faradayweg 4–6 14195 Berlin Germany

**Keywords:** carbon dioxide, catalytic reactions, mass spectrometry, methane, quantum chemistry calculations

## Abstract

Catalytic co‐conversion of methane with carbon dioxide to produce syngas (2 H_2_+2 CO) involves complicated elementary steps and almost all the elementary reactions are performed at the same high temperature conditions in practical thermocatalysis. Here, we demonstrate by mass spectrometric experiments that RhTiO_2_^−^ promotes the co‐conversion of CH_4_ and CO_2_ to free 2 H_2_+CO and an adsorbed CO (CO_ads_) at room temperature; the only elementary step that requires the input of external energy is desorption of CO_ads_ from the RhTiO_2_CO^−^ to reform RhTiO_2_^−^. This study not only identifies a promising active species for dry (CO_2_) reforming of methane to syngas, but also emphasizes the importance of temperature control over elementary steps in practical catalysis, which may significantly alleviate the carbon deposition originating from the pyrolysis of methane.

## Graphical abstract

The catalytic co‐conversion of CH_4_ and CO_2_ to syngas mediated by single Rh atom‐doped titanium oxide anions (RhTiO_2_^−^) has been experimentally identified. The production of free 2 H_2_+CO and an adsorbed CO (CO_ads_) could be achieved at room temperature while the only elementary step that requires the input of external energy is desorption of the CO_ads_ from RhTiO_2_CO^−^.

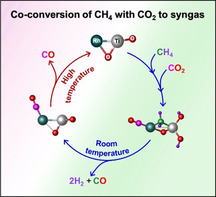

The production of chemical feedstocks from catalytic co‐conversion of methane and carbon dioxide, two abundant substances occurring in nature, reduces chemical industry's dependency on traditional fossil fuels and contributes to the mitigation of the greenhouse effect.^[1]^ Dry (CO_2_) reforming of methane^[2]^ [DRM, reaction 1] is a potential route to produce syngas (a mixture of carbon monoxide and hydrogen) that is a crucial feedstock for alcohols, olefins, and Fischer–Tropsch products.^[3]^ However, the inherent stability of both molecules, CH_4_ and CO_2_, as well as the highly endothermic nature of DRM to syngas requires this thermo‐catalysis to be run at high temperatures (*T*>1000 K), which inevitably leads to coke deposition and catalyst deactivation.^[4]^ Identifying each elementary step of a catalytic cycle offers the possibility to precisely optimize the reaction process and reduce energy consumption. However, catalytic DRM to syngas involves adsorption of CH_4_ and CO_2_, activation of four C−H bonds to dehydrogenate methane, cleavage of C=O bonds in CO_2_, H−H and C${}_{\mathrm{CH}{}_{4}}$ −O${}_{\mathrm{CO}{}_{2}}$ coupling, and desorption of two H_2_ and two CO molecules, making it experimentally challenging to follow each of the elementary steps in condensed phase studies.

(1) $$\mathrm{CH}{}_{4}+\mathrm{CO}{}_{2}\to 2\;\mathrm{CO}+2\;H{}_{2},\;\;\Delta H{}_{298}=+2.56\;\mathrm{eV}$$

Gas phase reactivity studies of isolated chemical species that compositionally resemble the local active sites of condensed phase catalysts provide a unique approach to probe the reaction intermediates and understand the molecular‐level details of elementary steps involved in the catalytic reactions of practical importance.^[5]^ While a great number of chemical entities (e.g., polyatomic clusters) have been experimentally discovered to activate CH_4_[^5f^, ^5g^, ^5h^, ^6^] or CO_2_[^5e^, ^7^] under specific reaction conditions, the number of identified species that promote the co‐conversion of CH_4_ and CO_2_ is very limited to date.^[8]^ The atomic cation Ta^+^ is able to co‐convert one CH_4_ and two CO_2_ molecules to H_2_+CO+C_2_H_2_O at room temperature, but the resulting TaO_2_^+^ is difficult to reduce to Ta^+^.^[8a]^ Although catalytic co‐conversion of CH_4_ and CO_2_ was later established over the bimetallic oxide anion RhVO_3_^−^, only co‐adsorption of CH_4_ and CO_2_ takes place at room temperature. An increase in the reaction temperature leads to efficient formation of methyl radicals.^[8c]^ Very recently, selective DRM was achieved at room temperature by using Rh_2_VO_1‐3_^−^ anions in combination with photoirradiation to produce the second H_2_ and CO molecules.^[8d]^ Herein, we demonstrate that RhTiO_2_^−^ co‐converts CH_4_ and CO_2_ to 2 H_2_+CO at room temperature without photo‐irradiation. The only elementary step that requires input of external energy to complete the catalytic cycle is the desorption of the second CO molecule.

Figures 1 and S1 show the mass spectra for the reactions of mass‐selected RhTiO_2_^−^ anions (**1**) with CH_4_ and CO_2_ in a linear ion trap reactor at room temperature under thermal collision conditions. Upon the interaction with 0.90 Pa CH_4_, at first for 1.6 ms, all of the RhTiO_2_^−^ anions were depleted and transformed to the adsorption product RhTiO_2_CH_4_^−^ [**2**, Figure 1 b and reaction 2a)]. Only very small amounts of RhTiO_2_CH_4_^−^ ions are dehydrogenated giving rise to the formation of RhTiO_2_CH_2_^−^ (**2**‐H_2_)+H_2_ [reaction 2b)]. The isotopic labeling experiment with CD_4_ (Figure S2) confirms the formation of the adsorption product, but the desorption of D_2_ molecules did not occur. The pseudo‐first (second) order rate constant (*k*_1_) for the reaction of RhTiO_2_^−^ with CH_4_ was determined as (2.2±0.4)×10^−11^ cm^3^ s^−1^ (Figure S2), corresponding to a reaction efficiency of (2.2±0.4)%.^[9]^ The kinetic isotopic effect (*k*_1_,${}_{\mathrm{CH}{}_{4}}$ /*k*_1_,${}_{\mathrm{CD}{}_{4}}$ ) was estimated to be 1.3±0.2.

(2a) $$\mathrm{RhTiO}{}_{2}{}^{-}+\mathrm{CH}{}_{4}\to \mathrm{RhTiO}{}_{2}\mathrm{CH}{}_{4}{}^{-}$$

(2b) $$\;\;\;\;\;\;\;\;\;\to \mathrm{RhTiO}{}_{2}\mathrm{CH}{}_{2}{}^{-}+H{}_{2}$$

**Figure 1:**
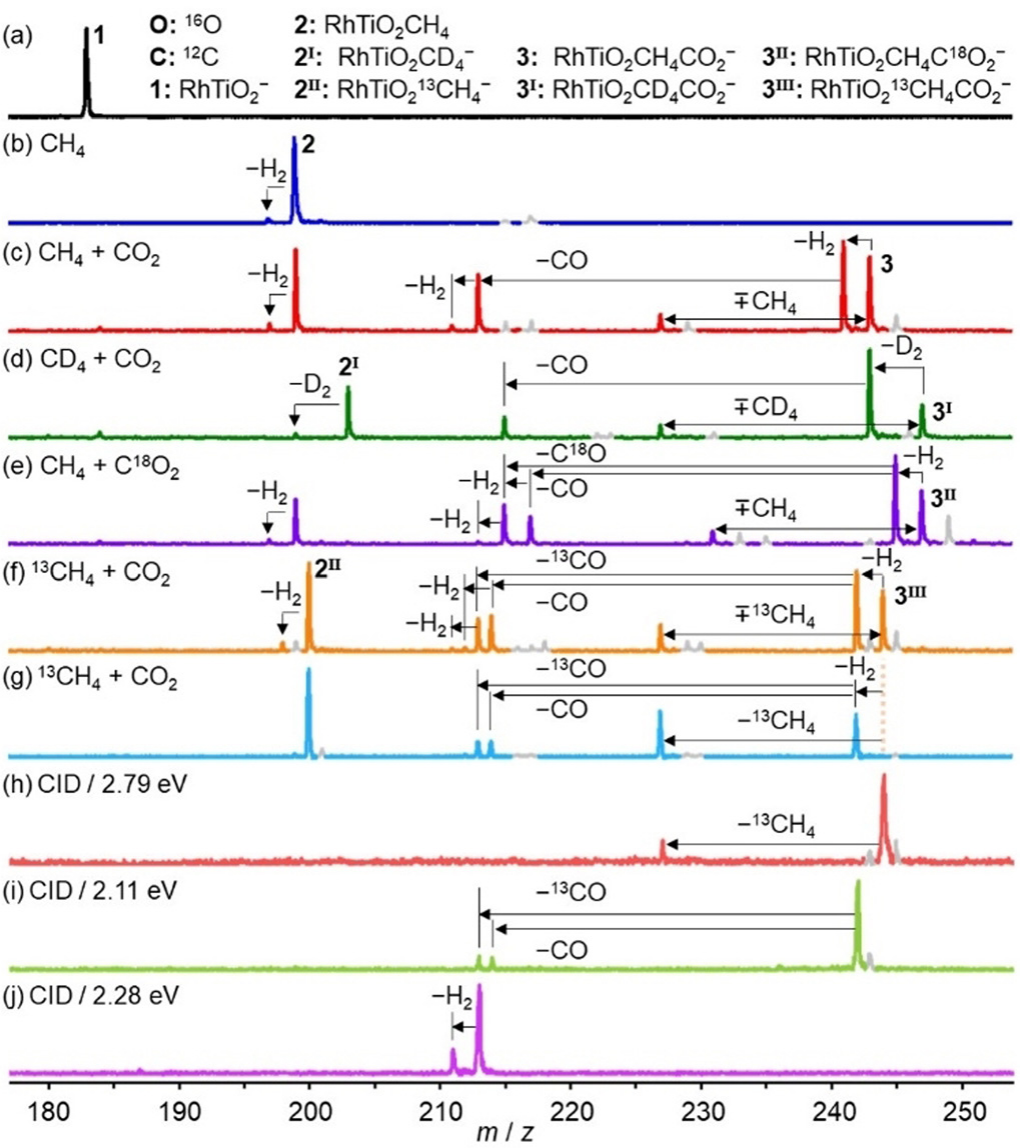
Mass spectra for the reactions of RhTiO_2_^−^ with He (a) and 0.90 Pa CH_4_ (b), RhTiO_2_CH_4_^−^ with 0.05 Pa CO_2_ (c), RhTiO_2_CD_4_^−^ with 0.03 Pa CO_2_ (d), RhTiO_2_CH_4_^−^ with 0.03 Pa C^18^O_2_ (e), and RhTiO_2_^13^CH_4_^−^ with 0.03 Pa CO_2_ (f) in the single ion trap experiment in which methane and carbon dioxide are delivered into the same ion trap. Panel (g) shows the mass spectrum for the reaction of mass‐selected product ion RhTiO_2_^13^CH_4_^−^ with 0.02 Pa CO_2_ in the double ion trap experiment in which methane and carbon dioxide are delivered into different ion traps. The arrows show the loss of neutral products from the ionic species or the adsorption of methane onto the ionic species. The reaction times are 1.6 ms for (b,g) and 2.5 ms for (c–f). The spectra for collision‐induced dissociation (CID) of RhTiO_2_^13^CH_4_CO_2_^−^ (h), RhTiO_2_^13^CH_2_CO_2_^−^ (i), and RhTiO_2_CH_2_O^−^ (j) generated from the reactions of RhTiO_2_^−^ with ^13^CH_4_/CH_4_ and CO_2_ in the same ion trap are shown in the bottom three panels in which the center‐of‐mass collisional energies are shown.

After all of the RhTiO_2_^−^ anions were converted to RhTiO_2_CH_4_^−^, CO_2_ molecules were pulsed into the ion trap and several new product signals appeared. Figure 1 c reveals that in addition to the co‐adsorption complex RhTiO_2_CH_4_CO_2_^−^ (**3**), a strong peak assigned to RhTiO_2_CH_2_CO_2_^−^ (P1) was observed, corresponding to the loss of the first H_2_ molecule [reaction 3a)]. A CO molecule can desorb from P1 to form an appreciable amount of RhTiO_2_CH_2_O^−^ ions [P2=P1−CO, reaction 3b)]. The detection of RhTiO_2_CO^−^ (P3=P2−H_2_) with relatively weak intensity implies that desorption of the second H_2_ molecule from P2 [reaction 3c)] is possible, but less efficient. Summarizing, RhTiO_2_^−^ promotes the co‐conversion of CH_4_ and CO_2_ to two free H_2_ molecules, one free CO molecule (2 H_2_+CO, syngas) at room temperature. Note that a product peak assigned to RhTiO_2_CO_2_^−^ generated from the CH_4_/CO_2_ exchange was also observed [reaction 3d)]. Isotopic labeling experiments with CD_4_ (Figure 1 d) confirm the reaction channels (3a)–3d).

(3a) $$\mathrm{RhTiO}{}_{2}\mathrm{CH}{}_{4}{}^{-}+\mathrm{CO}{}_{2}\to \mathrm{RhTiO}{}_{2}\mathrm{CH}{}_{2}\mathrm{CO}{}_{2}{}^{-}+H{}_{2}$$

(3b) $$\;\;\;\;\;\;\;\;\;\;\;\to \mathrm{RhTiO}{}_{2}\mathrm{CH}{}_{2}O{}^{-}+H{}_{2}+\mathrm{CO}$$

(3c) $$\;\;\;\;\;\;\;\;\;\;\;\to \mathrm{RhTiO}{}_{2}\mathrm{CO}{}^{-}+2\;H{}_{2}+\mathrm{CO}$$

(3d) $$\;\;\;\;\;\;\;\;\;\;\;\to \mathrm{RhTiO}{}_{2}\mathrm{CO}{}_{2}{}^{-}+\mathrm{CH}{}_{4}$$

(4) $$\mathrm{RhTiO}{}_{2}\mathrm{CO}{}_{2}{}^{-}+\mathrm{CH}{}_{4}\to \mathrm{RhTiO}{}_{2}\mathrm{CH}{}_{4}\mathrm{CO}{}_{2}{}^{-}$$

To clarify the O‐ and C‐atom source for CO production, additional isotopic labeling experiments with C^18^O_2_ and ^13^CH_4_ were conducted. Identification of the product ions RhTi^16^O_2_CH_2_^18^O^−^ and RhTi^16^OCH_2_^18^O_2_^−^ upon the interaction of RhTiO_2_CH_4_^−^ with C^18^O_2_ (Figure 1 e) shows that the O atom in CO originates from either CO_2_ or RhTiO_2_^−^. Next to the product ion RhTiO_2_^13^CH_2_^12^CO_2_^−^, two new signals of RhTiO_2_^12^CH_2_O^−^ and RhTiO_2_^13^CH_2_O^−^ were observed in the reaction of RhTiO_2_^13^CH_4_^−^ with CO_2_ (Figure 1 f), indicating that both CH_4_ and CO_2_ can provide a carbon atom for CO generation. Moreover, the weak peaks corresponding to RhTi^16^O_2_C^18^O^−^, RhTiO_2_^12^CO^−^, and RhTiO_2_^13^CO^−^ in Figure 1 e,f confirm desorption of the second H_2_ molecule.

In order to confirm the proposed sequential desorption mechanism, we spatially separated the addition of the reactants using two ion trap reactors (instead one). Product ions RhTiO_2_^13^CH_4_^−^ were produced in the first reactor, mass‐selected and then interacted with a gas pulse of CO_2_ in the second ion trap reactor (Figure 1 g). These experiments unambiguously confirm the reactions (3a)–(3d). However, the RhTiO_2_^13^CH_4_CO_2_^−^ product was absent, implying that the co‐adsorption complexes (3, 3^I^, 3^II^, and 3^III^) observed in Figure 1 c–f originated from the methane adsorption onto RhTiO_2_CO_2_^−^/RhTiO_2_C^18^O_2_^−^ [reaction (4)]. The CID experiments of mass‐selected intermediate complexes RhTiO_2_^13^CH_4_CO_2_^−^ (3^III^, Figure 1 f), RhTiO_2_^13^CH_2_CO_2_^−^ (P1, Figure 1 f), and RhTiO_2_CH_2_O^−^ (P2, Figure 1 c) with Xe were also conducted. The loss of ^13^CO and CO molecules from P1 and the loss of H_2_ from P2 correspond to reaction channels (3b) and (3c), respectively. The desorption of ^13^CH_4_ molecule from 3^III^ supports reaction (4).

These experimental results demonstrate that CH_4_ and CO_2_ are co‐converted to 2 H_2_+CO at room temperature in the presence of RhTiO_2_^−^ and form RhTiO_2_CO^−^ as the product ion. The CID experiments of mass‐selected RhTiO_2_CO^−^ with Xe were carried out. When the center‐of‐mass collisional energy was increased to above 3 eV, the loss of the second CO and regeneration of RhTiO_2_^−^ were observed [Figure S3 and reaction 5]. Thus, a catalytic cycle involving DRM to syngas [reaction (1)] over RhTiO_2_^−^ was experimentally achieved. Note, no co‐conversion was observed when the reaction gases were applied to the ion trap in reverse order, first CO_2_ followed by CH_4_ (Figures S4 and S5).

(5) $$\mathrm{RhTiO}{}_{2}\mathrm{CO}{}^{-}\overset{\mathrm{CID}}{\underset{}{\to }}\mathrm{RhTiO}{}_{2}{}^{-}+\mathrm{CO}$$

The structure of the RhTiO_2_^−^ anion was characterized by photoelectron spectroscopy (PES) combined with density functional theory (DFT) calculations (Figure S6).^[10]^ The lowest‐lying isomer of RhTiO_2_^−^ is a singlet that has a three‐membered ring of Rh‐Ti‐O with a terminal oxygen (O_t_) atom connected to Ti. Due to the absence of a reactor coupled with our PES apparatus and the relatively low ion intensities of reaction products, the structures of RhTiO_2_CH_4_^−^, RhTiO_2_CH_2_CO_2_^−^, RhTiO_2_CH_2_O^−^, and RhTiO_2_CO^−^ were not experimentally characterized. The possible structures of these ionic speices were computationally determined.

The most favorable pathways for the reactions of RhTiO_2_^−^ with CH_4_ and RhTiO_2_CH_4_^−^ with CO_2_ are shown in Figures 2, S7–S11. Methane binds to the Rh atom of RhTiO_2_^−^ under activation of the first C−H bond by oxidative addition (I1→I2) and formation of the stable intermediate I2. This is followed by the cleavage of the Rh−O bond in I2, H‐atom transfer from the Rh atom to the O_t_ atom (I2→I3), activation of the second C−H bond by the Rh atom and formation of I4, which contains a Rh−H bond, an OH group, and a three‐membered Rh‐CH_2_‐Ti ring. Dehydrogenation of I4 to RhTiO_2_CH_2_^−^ is not feasible due to high activation energy barriers (1.24–1.28 eV of I4→TS13/TS15 in Figure S7) and the hot I4 is stabilized by collisions with the buffer gas (He), in agreement with the experimental observation that the RhTiO_2_CH_4_^−^ mass peak dominates the mass spectrum (Figure 1 b).

**Figure 2:**
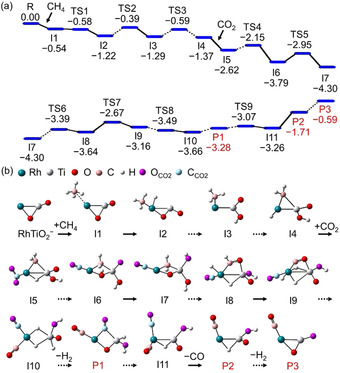
DFT‐calculated potential energy profile for the reaction of RhTiO_2_^−^+CH_4_+CO_2_ (R) reaction. The zero‐point vibration‐corrected energies with respect to the separated reactants (Δ*H*_0_) are given in eV. The structures of R, I1–I11, and P1–P3 are plotted and those of TS1–TS9 can be found in the Supporting Information.

The reaction of RhTiO_2_CH_4_^−^ (I4) with CO_2_ commences with the adsorption of CO_2_ and its activation involving the formation of a Rh−C bond in the encounter complex I5. Next, one of the C=O bonds is cleaved (I5→I22 and I23→I6, Figure S8); during this process, the H atom originally bound to Rh transfers to one of the O atoms (I22→I23 and I6→I7 in Figures 2 and S8), resulting in formation of the lowest energy structure, intermediate I7 (Figure S11), which consists of a four‐membered O‐Rh‐CH_2_‐Ti ring, two (Ti)−OH groups, and a (Rh)−CO moiety. One of the hydroxy H atoms then transfers back to Rh and H_2_C−O coupling occurs (I7→I24→I25 in Figure S8). Subsequently, the Rh−O bond is ruptured, followed by the successive activation of the third and the fourth C−H bond by the Rh atom (I25→I8→I9) and formation of I9 containing a Rh‐C‐O‐Ti moiety. After a series of structural rearrangements involving the CO moiety and H‐atom transfers (I9→I10), I10, which contains two (Rh)−CO moieties, two bridging and one terminal H atoms, is formed. After several more conversion steps, during which one of the CO moieties in I10 moves towards the Ti atom, molecular hydrogen is formed and evaporated to produce RhTiO_2_CH_2_CO_2_^−^ (P1, Δ*H*_0_=−3.28 eV).

P1 still possesses sufficient internal energy to enable desorption of the first CO molecule (P2, Δ*H*_0_=−1.71 eV) as well as the subsequent desorption of the second H_2_ molecule (P3, Δ*H*_0_=−0.59 eV; in the case that the RhTiO_2_CH_4_^−^ is not fully thermalized before reacting with CO_2_, the formation of P3 is possible). Note, the reaction course from P1 to P2 involves an important intermediate I11 that contains two equivalent CO moieties, [C_CH4_‐O_cluster_] and [C${}_{\mathrm{CO}{}_{2}}$ −O${}_{\mathrm{CO}{}_{2}}$ ], bound to the Rh atom. Either C${}_{\mathrm{CH}{}_{4}}$ O_cluster_ or (CO)${}_{\mathrm{CO}{}_{2}}$ can be desorbed to produce the ionic product RhTiO_2_CH_2_O^−^ (P2), consistent with the observed reaction channels in the isotopic labeling experiment (Figure 1). The reaction path for preferential evaporation of the second H_2_ molecule from P1 (P1→RhTiO_2_C_2_O_2_^−^+H_2_, Δ*H*_0_=−2.32 eV) was also considered. Although it is thermodynamically more favorable than P1→RhTiO_2_CH_2_O^−^+CO, a high energy barrier (1.18 eV of P1→TS49, in Figure S12) is encountered before H_2_ desorption. The rate (4.3×10^8^ s^−1^) of traversing TS49 from P1 was estimated to be two orders of magnitude lower than that for CO evaporation (I11→P2, 1.4×10^10^ s^−1^) so that CO is preferentially desorbed from P1. The RhTiO_2_C_2_O_2_^−^+H_2_ channel was not observed. After dehydrogenation of RhTiO_2_CH_2_O^−^ (P2→P3), the RhTiO_2_CO^−^ ion with CO bound to Rh is formed. Desorption of the second CO to regenerate RhTiO_2_^−^ and close the catalytic cycle of DRM to syngas is overall endothermic by 2.74 eV (Figure S13). This requires supplying additional external energy as shown by the CID experiments. The direct elimination of HCHO and CH_3_OH from the reaction of RhTiO_2_CH_4_^−^ with CO_2_ was also considered. These reaction pathways (Figures S14 and S15) are kinetically less favorable than the H_2_ loss channel [reaction (3a)] and ultimately also entropically unfavorable. Therefore, we conclude that the probability for formation of HCHO or CH_3_OH is negligible.

Methane conversion has been extensively explored in gas phase studies, which revealed that only a few transition metal‐containing oxide ions react with a single CH_4_ molecule to generate syngas at room temperature. These are the monometallic species ReO_3_^+[9]^ and RuO_3_^+[10]^ as well as the bimetallic systems RhAl_3_O_4_^+[11]^ and RhAl_2_O_4_^−^.^[12]^ However, only a single free H_2_ molecule was produced from one CH_4_ molecule, because the oxide ions commonly bind the remaining two H atoms in form of two hydroxy groups that are reluctant to liberate H atoms. While the situation is similar in RhTiO_2_^−^/CH_4_, i.e., the adsorption complex I4 contains an OH group (Figure 2), which makes H_2_ release unfavorable (**2**‐H_2_ in Figure 1 b). By sharp contrast, the introduction of CO_2_ into the reaction system enables the conversion of the four H atoms in CH_4_ to two free H_2_ molecules at room temperature, greatly enhancing the conversion efficiency of CH_4_ to H_2_. This work also confirms that by selecting a suitable oxide support (e.g. TiO_2_^−^ cluster), the single Rh atom is sufficiently active to enable the co‐conversion of methane with carbon dioxide to syngas, in sharp contrast to the VO_*x*_ cluster support, for which the Rh_2_ dimer is indispensible for syngas production.^[8d]^

In condensed phase thermocatalysis of DRM to syngas, methane activation is generally viewed as the rate‐determining step and almost all the elementary reactions are performed at the same high temperatures that easily caused pyrolysis of methane (CH_4_→C+2 H_2_), leading to carbon deposition and catalyst deactivation.^[2c]^ Considerable efforts have been devoted to reduction of carbon deposition by engineering the composition and morphology of catalysts.[^4a^, ^4c^] Our gas phase study on the thermocatalytic DRM to syngas on RhTiO_2_^−^ provides first experimental evidence that both the conversion of CH_4_ to 2 H_2,gas_+CO_gas_/CO_ads_ and reduction of CO_2_ to CO_ads_/CO_gas_ can be achievable at room temperature, the only elementary step that requires high temperatures is CO_ads_ desorption (rate‐determining step). The insights into the elementary steps of this study should motivate the employment of temperature‐programmed methods^[13]^ which could precisely engineer the reaction course of DRM at room or high temperature in practical catalysis. This may significantly alleviate the carbon deposition originating from the pyrolysis of methane.

In conclusion, the co‐conversion of CH_4_ and CO_2_ to syngas mediated by RhTiO_2_^−^ has been achieved in the gas phase. The experimental identification of multiple ionic reaction intermediates confirms that, in principle, the reaction of CH_4_+CO_2_→2 H_2,gas_+CO_gas_+CO_ads_ can occur at room temperature. This is also supported by quantum chemical calculations. Only the final step of CO_ads_ desorption requires the input of external energy (e.g., high temperature) to complete the full catalytic cycle. The working conditions for each elementary step of syngas production identified herein underline that the temperature control is very important for optimization of the reaction process and reduction of carbon deposition and energy consumption in practical catalysis.

## Conflict of interest

The authors declare no conflict of interest.

## Supporting information

As a service to our authors and readers, this journal provides supporting information supplied by the authors. Such materials are peer reviewed and may be re‐organized for online delivery, but are not copy‐edited or typeset. Technical support issues arising from supporting information (other than missing files) should be addressed to the authors.

Supplementary
