# Supplementary material for: Catalytic Co‐Conversion of CH4 and CO2 Mediated by Rhodium–Titanium Oxide Anions RhTiO2 −
Source: Angew Chem Int Ed Engl. 2021 May 17;60(25):13788–92. doi: 10.1002/anie.202103808 (PMC8251526; doi:10.1002/anie.202103808)
Supplement: Supplementary file 1 — Supplementary [file ANIE-60-13788-s001.pdf]

Angewandte Chemie
Eine Zeitschrift der Gesellschaft Deutscher Chemiker

Supporting Information

Catalytic Co-Conversion of CH_4 and CO_2 Mediated by Rhodium–Titanium Oxide Anions $RhTiO_2^-$

Yuan Yang, Ya-Ke Li, Yan-Xia Zhao, Gong-Ping Wei, Yi Ren, Knut R. Asmis,* and Sheng-Gui He**

anie_202103808_sm_miscellaneous_information.pdf

Contents

1. **Experimental method.** (Page S2)
2. **Theoretical method.** (Page S3)
3. **Additional mass spectra for cluster reactions.**

Figure S1. Assignment of the grey peaks in Figure 1 in the main text. (Pages S4 and S5)
Figure S2. TOF mass spectra for the reactions of $RhTiO_2^-$ + CH_4/CD_4 and kinetic analysis. (Page S6)
Figure S3. Collision induced dissociation of $RhTiO_2CO^-$ cluster. (Page S7)
Figure S4. TOF mass spectra for the reaction of $RhTiO_2^-$ + CO_2 and kinetic analysis. (Page S7)
Figure S5. TOF mass spectra for the reaction of $RhTiO_3^-$ + CH_4. (Page S8)

4. **Additional results for cluster structures and reaction pathways.**

Figure S6. Structural assignment of $RhTiO_2^-$ cluster. (Page S9)
Figure S7. Potential energy profile for the reaction of $RhTiO_2^-$ + CH_4. (Page S10)
Figure S8. Potential energy profile for the reaction of $RhTiO_2CH_4^-$ (I4) + CO_2 to P1-P3. (Page S11)
Figure S9. Potential energy profile for the reaction of $RhTiO_2CH_4^-$ (I4) + CO_2 → $RhTiO_2CO_2^-$ + CH_4 and the reaction of $RhTiO_2CO_2^-$ + CH_4→ $RhTiO_2CH_4CO_2^-$. (Page S12)
Figure S10. Potential energy profile for the reaction of $RhTiO_2CH_4^-$ (I2/I3) + CO_2 → I7. (Page S13)
Figure S11. Isomeric structures of $RhTiO_2CH_4CO_2^-$ species. (Page S14)
Figure S12. Potential energy profile for the reaction of $RhTiO_2C_2H_2O_2^-$ (P1) → $RhTiO_2C_2O_2^-$ + H_2. (Page S15)
Figure S13 Thermodynamic data for the elementary reactions of DRM to syngas catalyzed by $RhTiO_2^-$ anion. (Page S16)
Figure S14. A comparison of the potential energy profiles for the formations of HCHO versus H_2 + CO from $RhTiO_2^-$ + CH_4 + CO_2. (Page S17)
Figure S15. A comparison of the DFT calculated potential energy profiles for the formations of CH_3OH versus $2H_2$ + CO from $RhTiO_2^-$ + CH_4 + CO_2 (Page S18)
Figure S16. Two different mechanisms of DRM to syngas over $RhTiO_2^-$ (Page S19)
Figure S17. Potential energy profile for the coupling of C_{CH4} and O_{CO2}. (Page S20)

5. **References** (Page S21)

1. Experimental Method.

The negatively charged rhodium-titanium bimetallic oxide clusters ($Rh_xTi_yO_z^-$) were generated by laser ablation of a rotating and translating metal disk compressed with Rh and ^{48}Ti powders (molar ration of 2:1) in the presence of 0.4% O_2 seeded in a helium carrier gas with the backing pressure of 6.0 standard atmospheres. Among many tested $Rh_xTi_yO_z^-$ clusters, the $RhTiO_2^-$ cluster ions of interest were mass-selected using a quadrupole mass filter (QMF)[1] and entered into a linear ion trap (LIT) reactor[2], where they were confined and thermalized by collisions with a pulse of buffer gas He and then reacted with $^{12}CH_4$, $^{13}CH_4$, CD_4, $C^{16}O_2$ or $C^{18}O_2$. The $RhTiO_2CH_4^-$, $RhTiO_2{}^{13}CH_4^-$, and $RhTiO_2CD_4^-$ ions were pre-produced by seeding 20% CH_4, $^{13}CH_4$ and CD_4 in the He buffer gas to react with the $RhTiO_2^-$ cluster ions in the LIT, respectively. The generated $RhTiO_2CH_4^-$, $RhTiO_2{}^{13}CH_4^-$ or $RhTiO_2CD_4^-$ ions then reacted with 5% $C^{16}O_2$ or 20% $C^{18}O_2$ seeded in He gas for about 2.5 ms in the LIT. A reflection time-of-flight mass spectrometer[3] (TOF-MS) was used to detect the cluster ions ejected from the LIT reactor.

The rate constants of the reactions between $RhTiO_2^-$ cluster and CH_4, CD_4, or CO_2 were determined by using the following equations:

$$I_\mathrm{R} = \exp(-k_1 \times \rho \times t_\mathrm{R}) \qquad \text{(S1a)}$$

$$I_\mathrm{P} = 1\text{-}\exp(-k_1 \times \rho \times t_\mathrm{R}) \qquad \text{(S1b)}$$

in which I_R and I_p are the relative intensities of the reactant and product cluster ions, respectively; k_1 is the (second order) rate constant of a pseudo-first-order reaction, ρ is the molecular density of CH_4, CD_4, or CO_2 in the ion trap reactor, and t_R is the reaction time.

The collision-induced dissociation (CID) experiments of $RhTiO_2CO^-$ were performed by introducing xenon into the LIT reactor (run at the collision cell mode) for collisions with $RhTiO_2CO^-$ clusters ions, of which the translational energies could be fixed at different values. The $RhTiO_2CO^-$ ions were generated by laser ablation in the presence of an O_2/CO gas mixture seeded in a helium carrier gas. The pressure of Xe in the LIT was low (60 mPa) for single collision conditions (the average number of collisions was estimated to be 0.08). The collision energies between a cluster ion and an Xe atom were converted from the laboratory frame (E_lab) to center-of-mass frame (E_cm) by using $E_\mathrm{cm} = E_\mathrm{lab} \times m/(m + M)$, in which M and m are the masses of the cluster ion and Xe, respectively.

In addition to the above reactivity and CID experiments in which a single ion trap system was used, the experiments by using a newly-developed double ion trap system were carried out. The double ion trap system includes two QMFs and two LITs. The first set of QMF/LIF was used for the reaction of mass-selected $RhTiO_2^-$ with CH_4 or gas mixtures of CH_4/CO_2 and the second QMF can mass-select the product ions from the first LIT to inject into the second LIT for further reaction with CO_2 molecule or collision with Xe atom, respectively. In such experiments, the reactant cluster ions of interest ($RhTiO_2^-$) were generated in the same way as in the single ion trap experiments. The conditions to run the second LIT for the reaction (with CO_2) and the collision (with Xe) are similar to those used in the corresponding single ion trap experiment.

The photoelectron imaging spectroscopy (PEIS) of $RhTiO_2^-$ was studied with a separated vacuum system. The generated $RhTiO_2^-$ was selected by a mass gate and crossed with a 355 nm or 425 nm laser beam. The electrons from photo-detachment were energy-analyzed by the photoelectron imaging spectrometer.[4] The PEIS spectrum was calibrated using the spectrum of Au^- taken at the similar conditions. The resolution of the photoelectron imaging spectrometer was approximately 30 meV at electron kinetic energy of 1 eV.

2. Theoretical Method.

The density functional theory (DFT) calculations using Gaussian 09 program[5] were carried out to investigate the structures of reactant cluster $RhTiO_2^-$, reaction complex $RhTiO_2X^-$ (X= CH_4, CH_4CO_2, CO), and products as well as the reaction pathways of $RhTiO_2^-$ with CH_4 and $RhTiO_2CH_4^-$ with CO_2. The TPSS functional[6] has been proved to perform well for many Rh-doped metal oxide systems,[7,8] so the results by TPSS method are given throughout this work. The TZVP basis sets[9] for C, H, O, and Ti atoms and the D95V basis set combined with the Stuttgart/Dresden relativistic effective core potentials (denoted as SDD in Gaussian software)[10] for Rh atom were used. The reaction pathways calculations involved geometry optimization of reaction intermediates (IMs) and transition states (TSs) through which the IMs transfer to each other. The initial guess structures of the TS species were obtained through relaxed potential energy surface scans using single or multiple internal coordinates.[11] Vibrational frequency calculations were performed to check that the IMs or TSs have zero and only one imaginary frequency, respectively. Intrinsic reaction coordinate calculations were performed so that a transition state connects two appropriate local minima. The zero-point vibration corrected energies (ΔH_0) in unit of eV are reported in this work.

3. Additional mass spectra for cluster reactions.

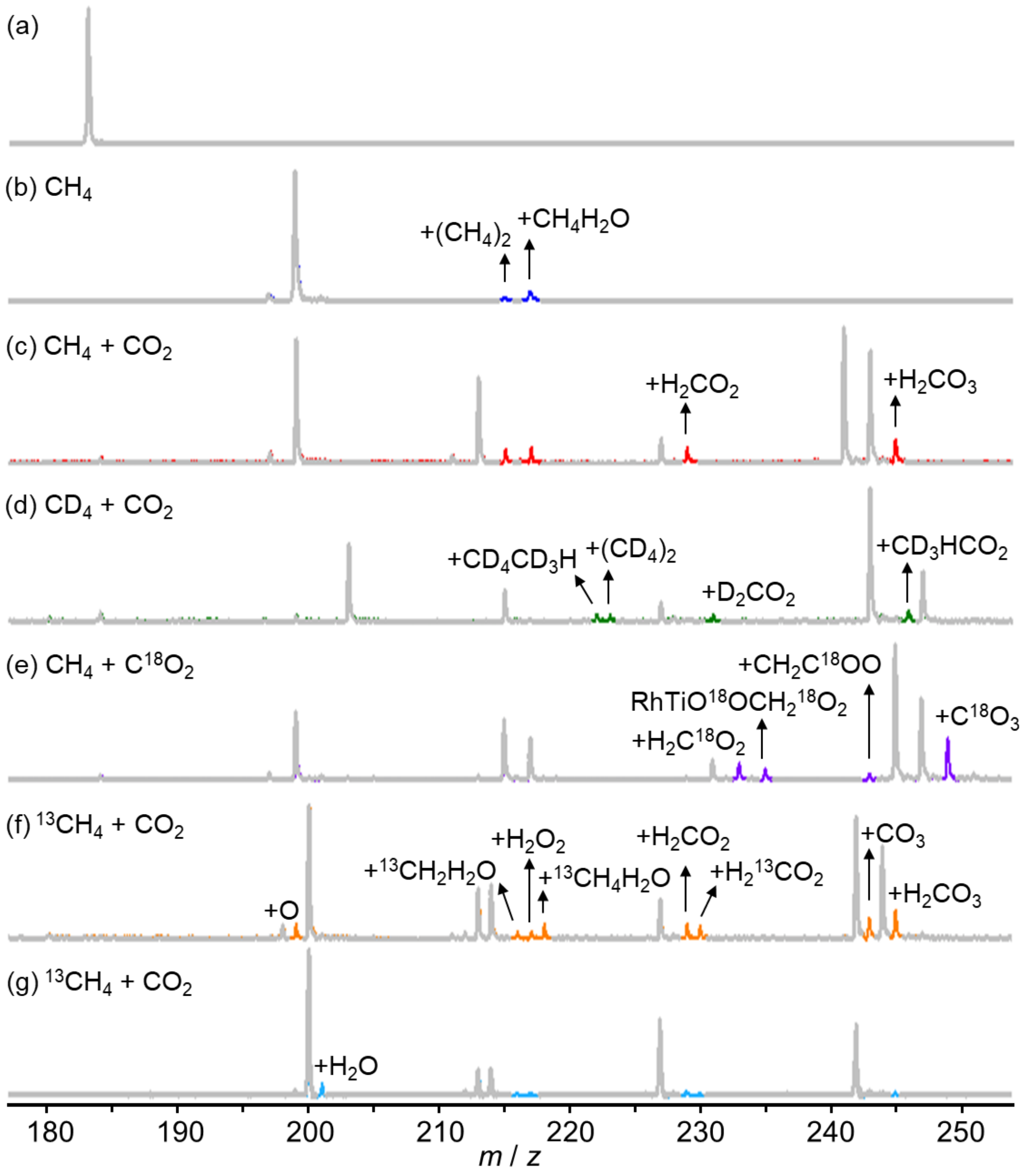

Figure S1. Assignment of the grey peaks in Figure 1. These minor peaks are due to the reactions with the second CH_4 or second CO_2 molecule, and with the residual H_2O in the LIT. The $RhTiO_2X^-$ (X= O, H_2O, etc.) species are labeled as +X (see Figure 1 in the main text for the assignment of unlabeled peaks).

The minor peaks can be formed by the following reactions.

Figure S1b:

$RhTiO_2CH_4^- + CH_4 \rightarrow RhTiO_2(CH_4)_2^-$

$RhTiO_2CH_4^- + H_2O \rightarrow RhTiO_2CH_4H_2O^-$

Figure S1c:

$RhTiO_2H_2CO^- + CO_2 \rightarrow RhTiO_2H_2CO_2^- + CO$

$RhTiO_2H_2CO_2^- + CO_2 \rightarrow RhTiO_2H_2CO_3^- + CO$

Figure S1d:

$RhTiO_2CD_4^- + CD_4 \rightarrow RhTiO_2(CD_4)_2^-$

$RhTiO_2(CD_4)_2^- + H_2O \rightarrow RhTiO_2CD_4CD_3H^- + HDO$

$RhTiO_2D_2CO^- + CO_2 \rightarrow RhTiO_2D_2CO_2^- + CO$

$RhTiO_2CD_4CO_2^- + H_2O \rightarrow RhTiO_2CD_3HCO_2^- + HDO$

Figure S1e:

$RhTiO_2H_2C^{18}O^- + C^{18}O_2 \rightarrow RhTiO_2H_2C^{18}O_2^- + C^{18}O$

$RhTiO^{18}OH_2C^{18}O^- + C^{18}O_2 \rightarrow RhTiO^{18}OH_2C^{18}O_2^- + C^{18}O$

$RhTiO_2CH_2C^{18}O_2^- + H_2O \rightarrow RhTiO_2CH_2C^{18}OO^- + H_2{}^{18}O$

$RhTiO_2C^{18}O_2^- + C^{18}O_2 \rightarrow RhTiO_2C^{18}O_3^- + C^{18}O$

Figure S1f:

$RhTiO_2^- + H_2O \rightarrow RhTiO_3^- + H_2$

$RhTiO_2{}^{13}CH_2^- + H_2O \rightarrow RhTiO_2{}^{13}CH_2H_2O^-$

$RhTiO_3^- + H_2O \rightarrow RhTiO_2H_2O_2^-$

$RhTiO_2{}^{13}CH_4^- + H_2O \rightarrow RhTiO_2{}^{13}CH_4H_2O^-$

$RhTiO_2H_2CO^- + CO_2 \rightarrow RhTiO_2H_2CO_2^- + CO$

$RhTiO_2H_2{}^{13}CO^- + CO_2 \rightarrow RhTiO_2H_2{}^{13}CO_2^- + CO$

$RhTiO_2CO_2^- + CO_2 \rightarrow RhTiO_2CO_3^- + CO$

$RhTiO_2CO_2^- + H_2O \rightarrow RhTiO_2H_2CO_3^-$

Figure S1g:

$RhTiO_2{}^{13}CH_4^- + H_2O \rightarrow RhTiO_2H_2O^- + {}^{13}CH_4$

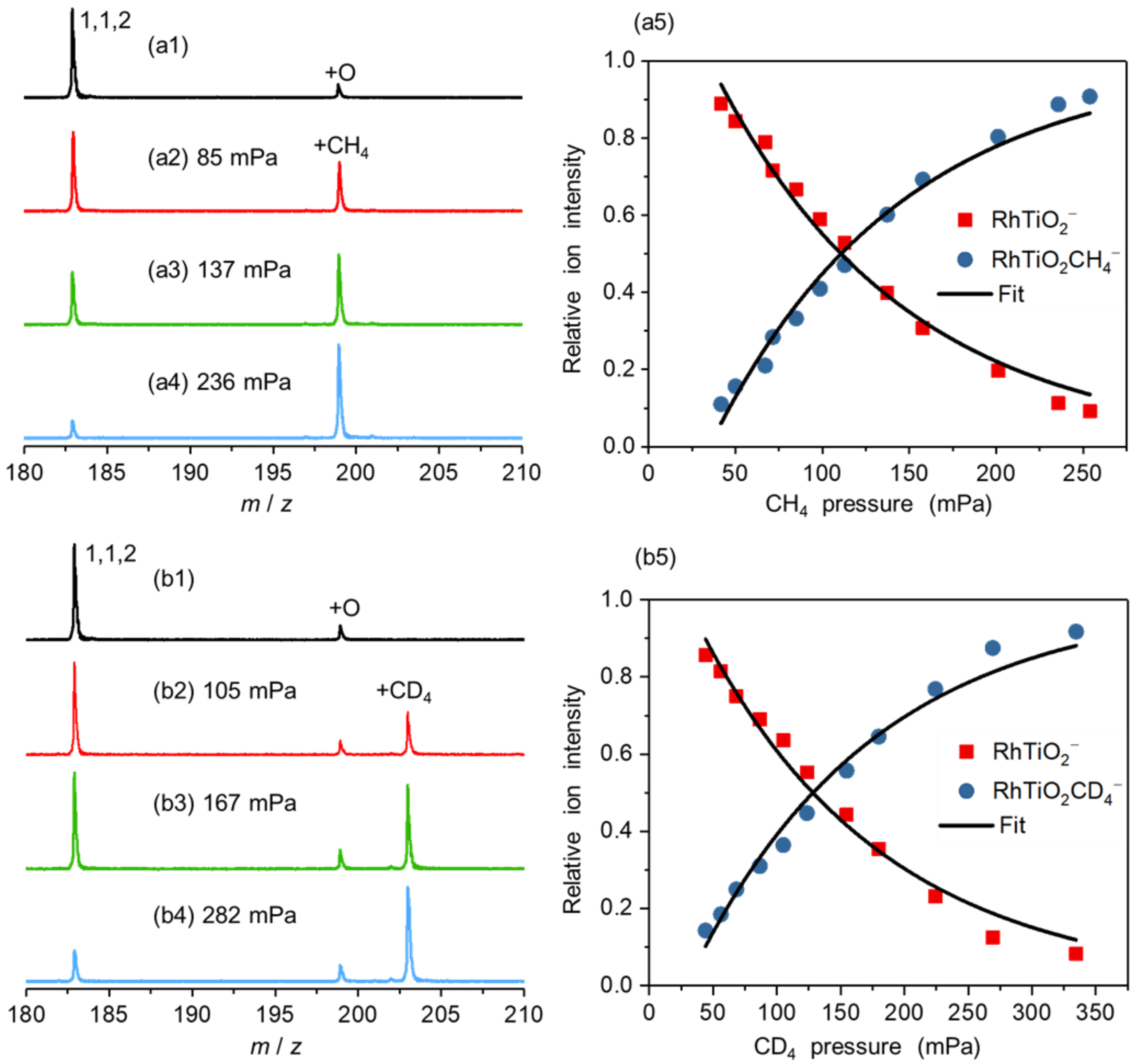

Figure S2. TOF mass spectra for the reactions of $RhTiO_2^-$ cluster with CH_4 (a1-a4) and CD_4 (b1-b4) at 298 K. The reactant gas pressures are shown. The $RhTiO_2X^-$ (X = CH_4, CD_4. etc.) species are labeled as +X. The pannels of (a5) and (b5) plot the signal variation of the reactant and product ions with respect to the CH_4 and CD_4 pressure, respectively. The solid lines were fitted to the experimental data by using equations (S1a) and (S1b).

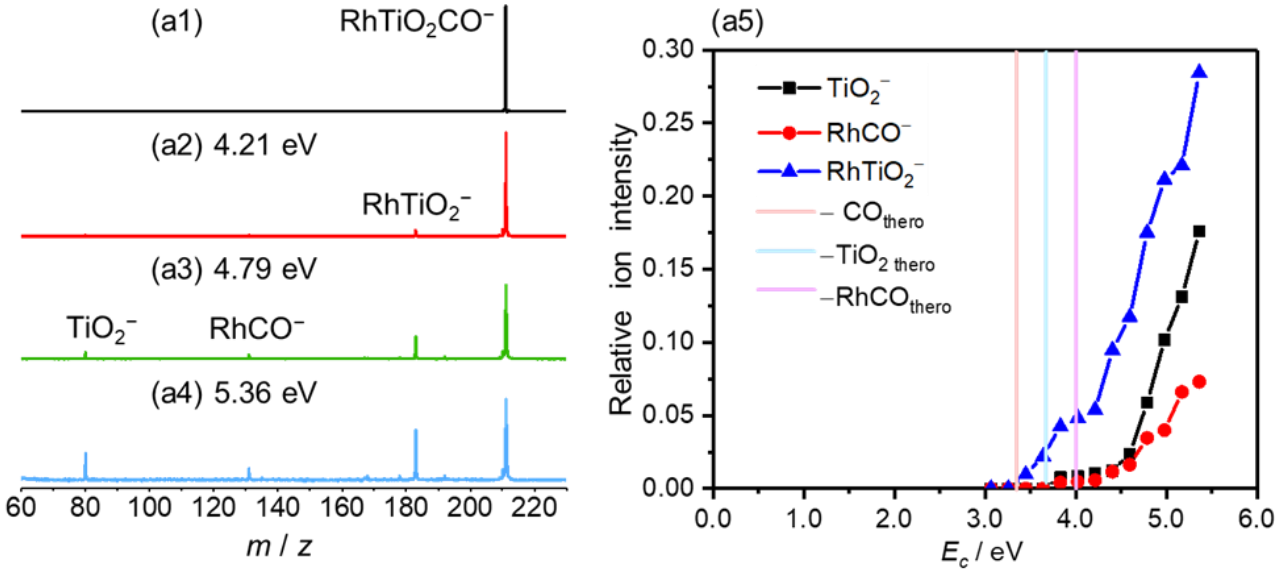

Figure S3. (a1-a4) The collision-induced dissociation spectra of $RhTiO_2CO^-$ with Xe at different center-of-mass collisional energies (E_c); (a5) The variation of relative intensities of the parent and daughter ions with respect to the E_c values. The vertical lines correspond to the theoretically predicted dissociation energies of $RhTiO_2CO^-$ (Figure S8, P3) → $RhTiO_2^-$ + CO (ΔH_0 = +3.33 eV), $RhTiO_2CO^-$ → $RhCO^-$ + TiO_2 (ΔH_0 = +3.70 eV), and $RhTiO_2CO^-$ → TiO_2^- + RhCO (ΔH_0 = +3.97 eV).

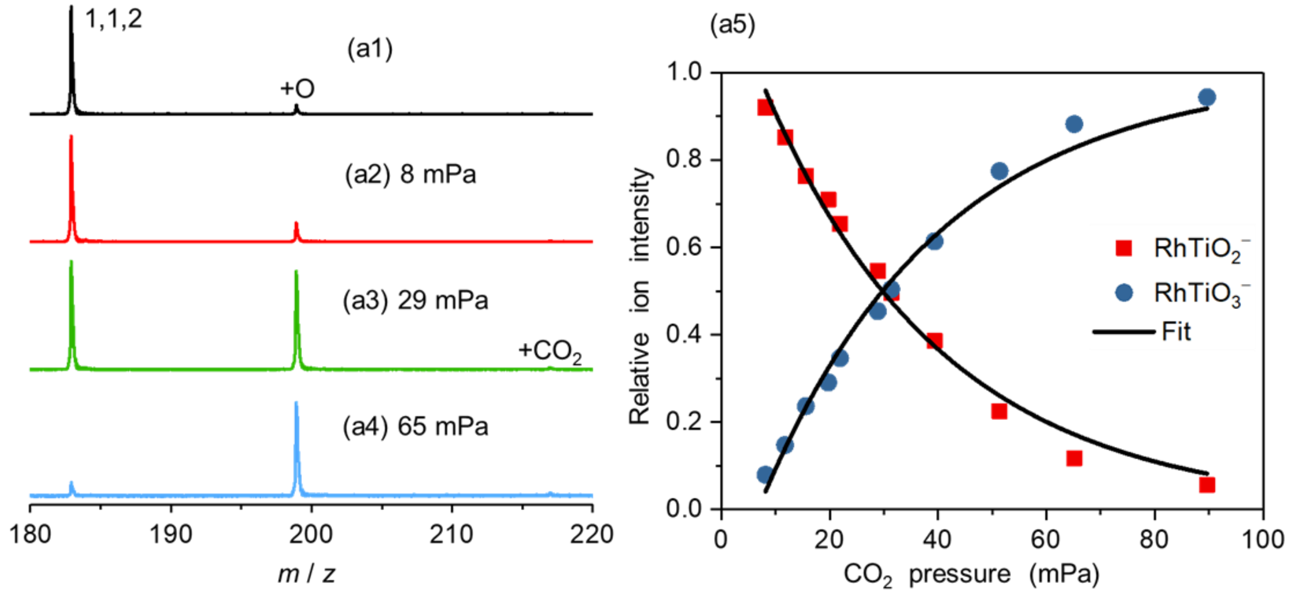

Figure S4. (a1-a4) TOF mass spectra for the reactions of $RhTiO_2^-$ cluster with CO_2 at 298 K. The reactant gas pressures are shown. The $RhTiO_2X^-$ (X = O, CO_2) species are labeled as +X. The pannel of (a5) plots the signal variation of the reactant and product ions with respect to the CO_2 pressure. The solid lines were fitted to the experimental data by using equations (S1a) and (S1b).

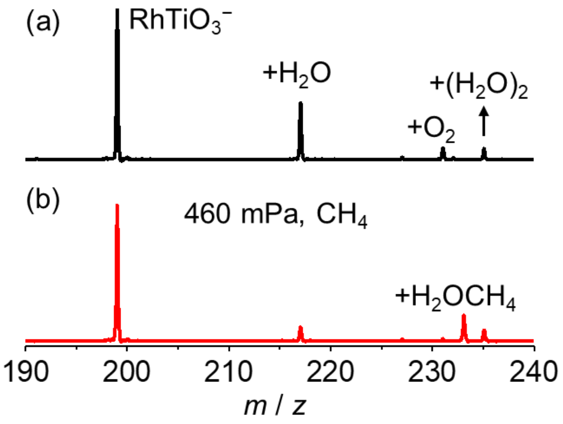

Figure S5. TOF mass spectra for the reaction of $RhTiO_3^-$ cluster with CH_4 at 298 K. The reactant gas pressure is shown. The $RhTiO_2X^-$ (X = H_2O, O_2. etc.) species are labeled as +X.

4. Additional results for cluster structures and reaction pathways.

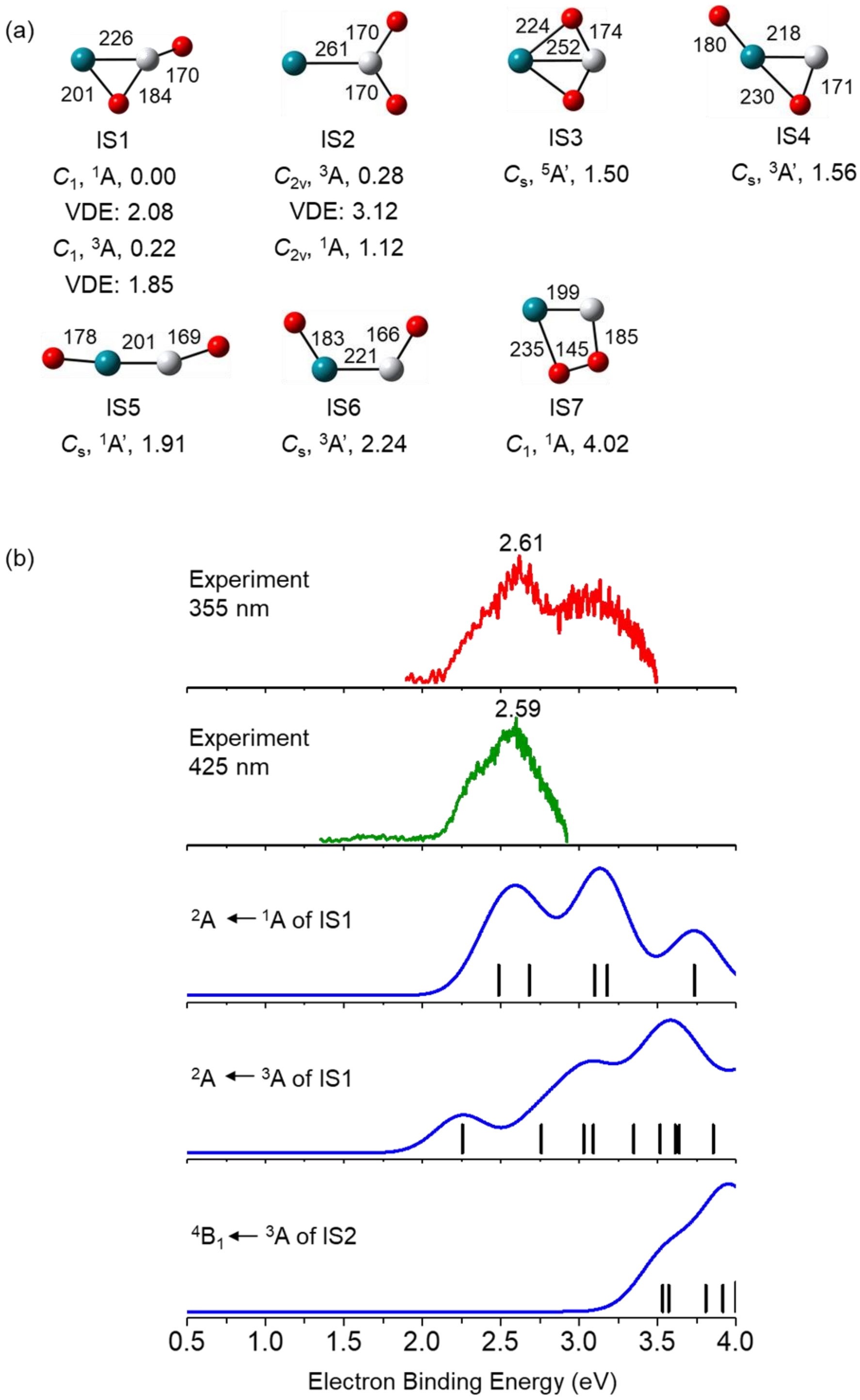

Figure S6. (a) DFT optimized isomeric structures of $RhTiO_2^-$ cluster. The symmetries and electronic states are shown. The superscripts are spin multiplicities. The zero−point vibration−corrected energies (ΔH_0) are given in eV. The vertical electron detachment energies (VDEs) of IS1 and IS2 are shown. (b) Comparison between the experimental photoelectron spectrum and the simulated density of states (DOS) spectra for the low-lying isomers of $RhTiO_2^-$. The DOS spectra were conducted by fitting the distribution of the transition lines with unit-area Gaussian functions of 0.30 eV full width at half maximum (FWHM). The DOS spectra for ^{1}A of IS1, ^{3}A of IS1, and ^{3}A of IS2 are blue-shifted by 0.41 eV.

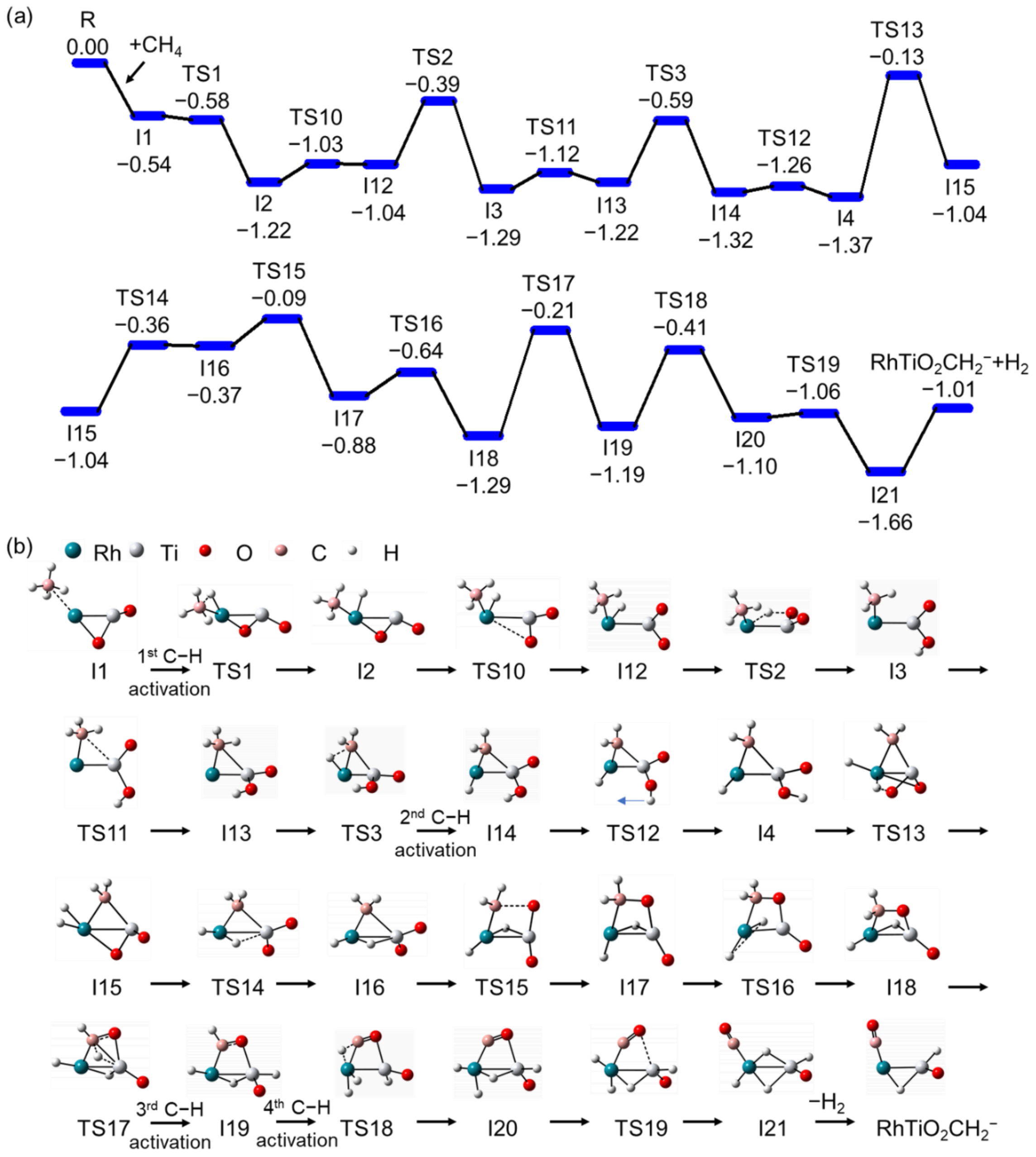

Figure S7. DFT calculated the most favorable potential energy profile for the reaction of $RhTiO_2^-$ (^1A of IS1) with CH_4. The zero−point vibration−corrected energies (ΔH_0, in unit of eV) of the reaction intermediates, transition states, and products with respect to the separated reactants ($RhTiO_2^-$ + CH_4) are given.

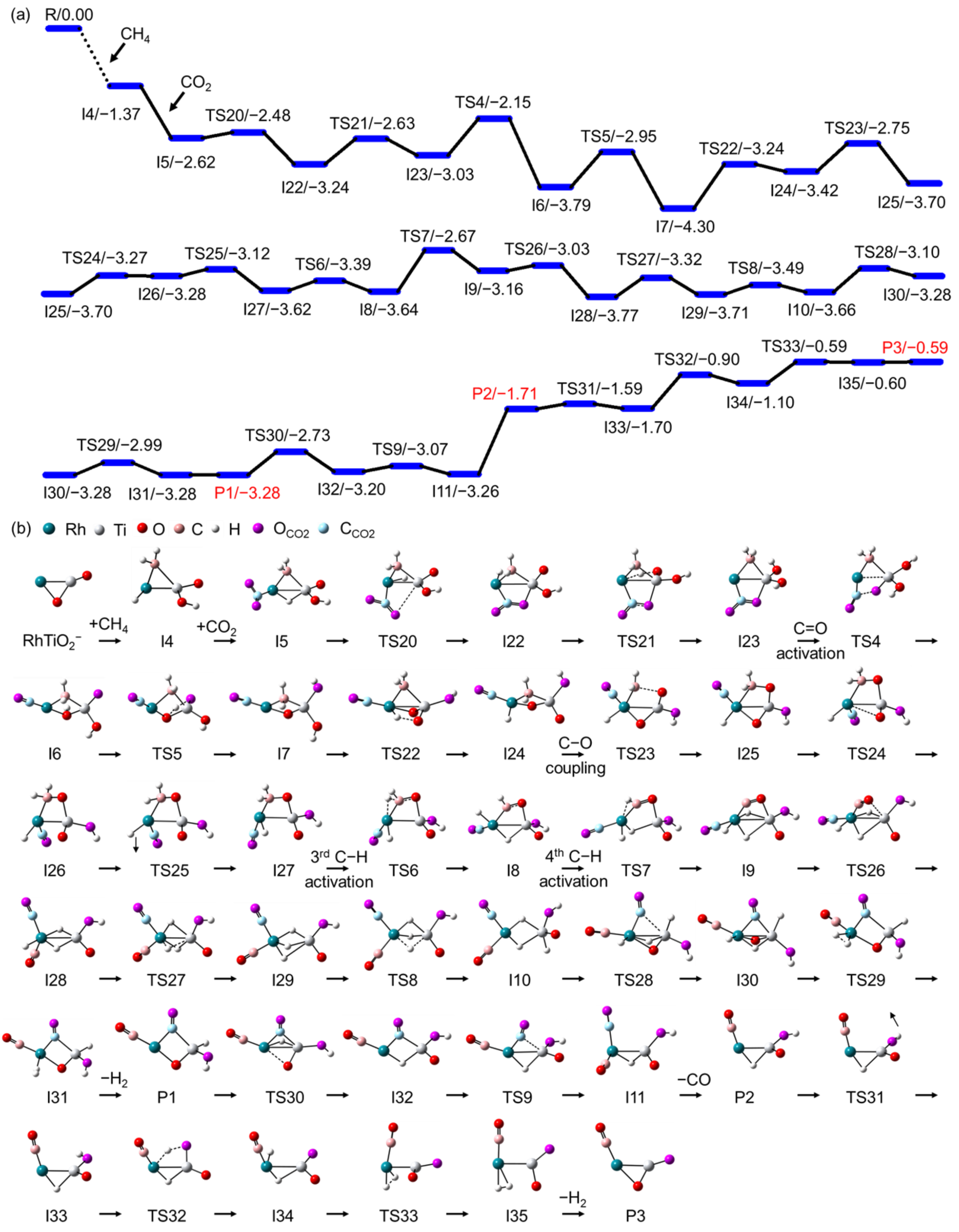

Figure S8. DFT calculated potential energy profile for the reaction of $RhTiO_2CH_4^-$ (I4) with CO_2. The zero−point vibration−corrected energies (ΔH_0, in unit of eV) of the reaction intermediates, transition states, and products with respect to the separated reactants ($RhTiO_2^-$ + CH_4 + CO_2) are given.

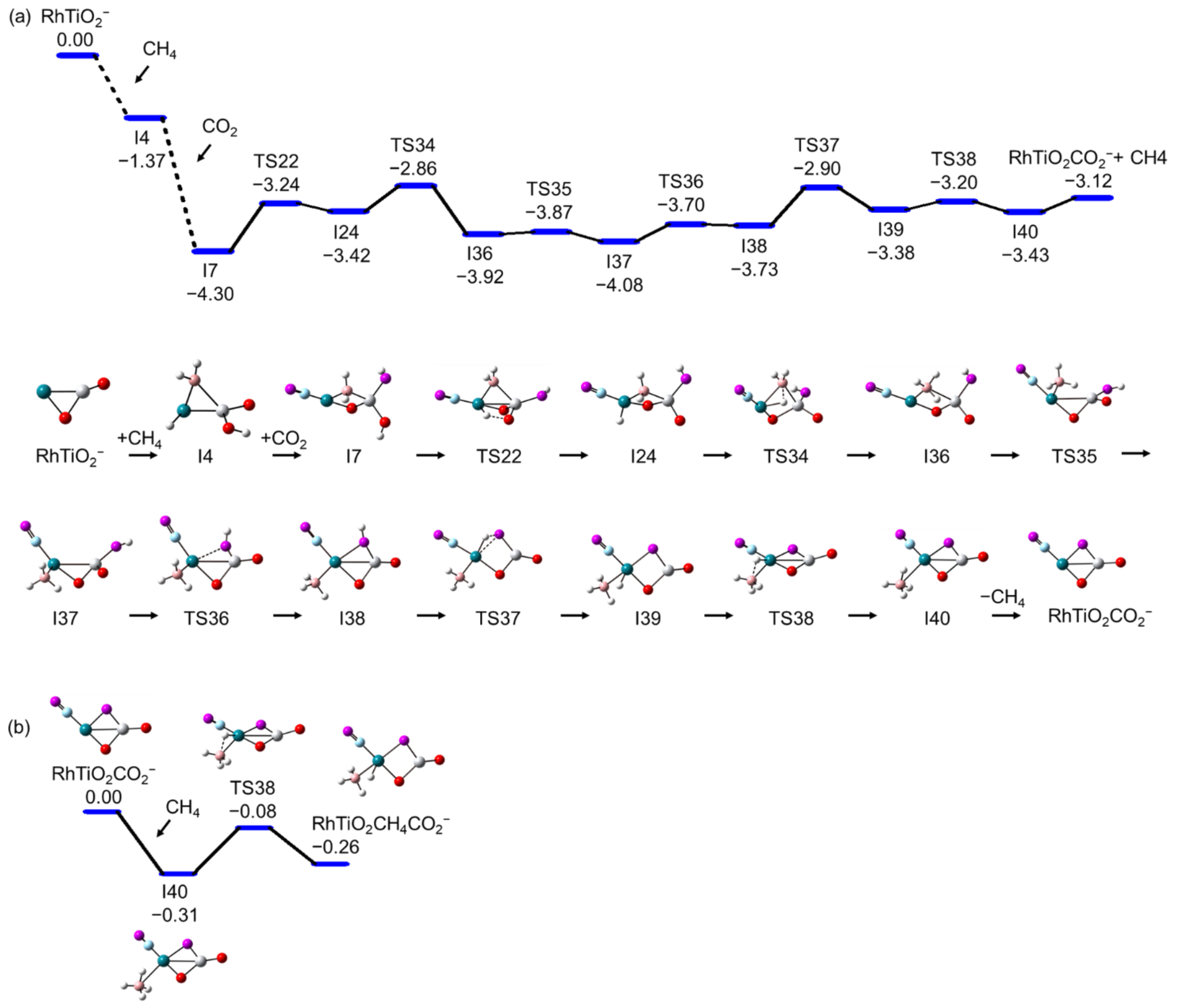

Figure S9. DFT calculated Potential energy profile for the reaction of $RhTiO_2CH_4^-$ (I4) + CO_2 → $RhTiO_2CO_2^-$ + CH_4 (a) and the reaction of $RhTiO_2CO_2^-$ + CH_4→ $RhTiO_2CH_4CO_2^-$ (b). The zero−point vibration−corrected energies (ΔH_0, in unit of eV) of the reaction intermediates, transition states, and products with respect to the separated reactants ($RhTiO_2^-$ + CH_4 + CO_2) are given.

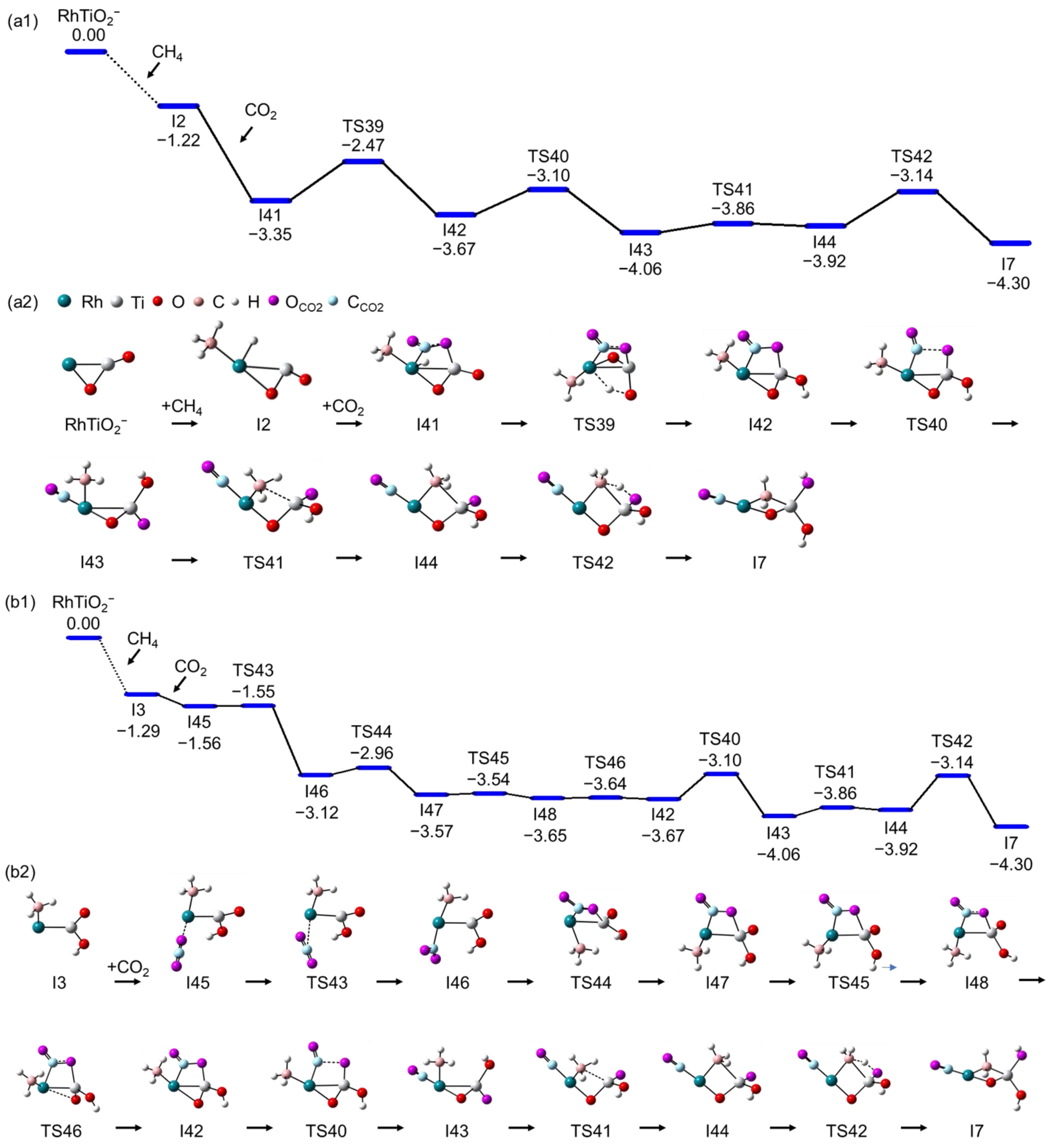

Figure S10. DFT calculated potential energy profiles for the formation of I7 (the most stable species in Figure 3 of the main text) from $RhTiO_2CH_4^-$(I2, a) + CO_2 and $RhTiO_2CH_4^-$(I3, b) + CO_2. The relative energies (ΔH_0, eV) of the reaction intermediates, transition states, and products with respect to the separated reactants ($RhTiO_2^-$ + CH_4 + CO_2) are given.

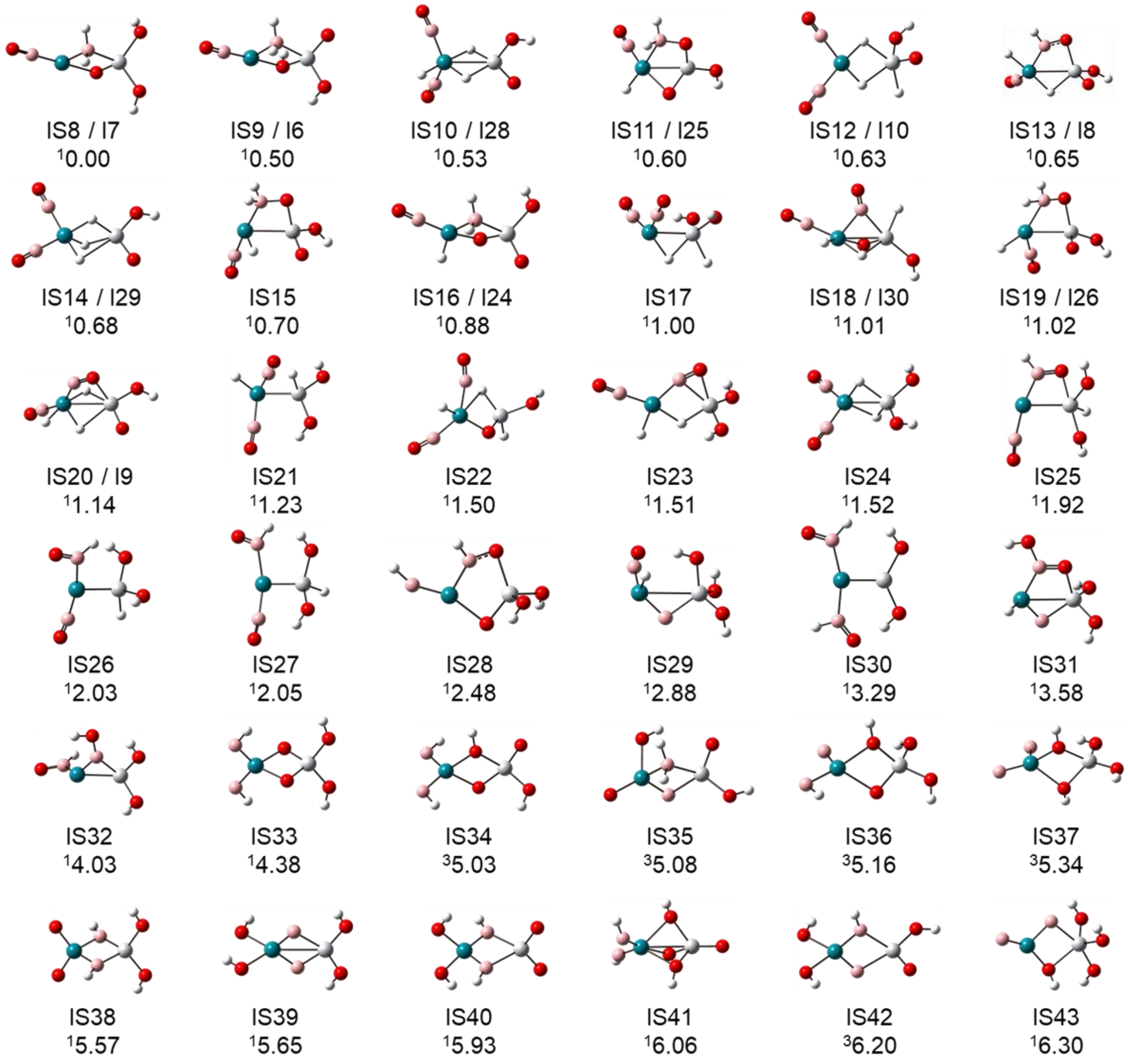

Figure S11. DFT optimized isomeric structures and relative energies (ΔH_0, in unit of eV) of $RhTiO_2CH_4CO_2^-$ species. The superscripts are spin multiplicities. The relative energy of the lowest-energy isomer (IS8) with respect to the separated reactants of $RhTiO_2^-$ + CH_4 + CO_2 is −4.30 at TPSS level. The isomers occur in the potential energy profiles such as those in Figures 3 and S8 are indicated: for example, IS8 is the same as I7 (the most stable species in Figure 3) and IS14 is the same as I29 in Figure S8.

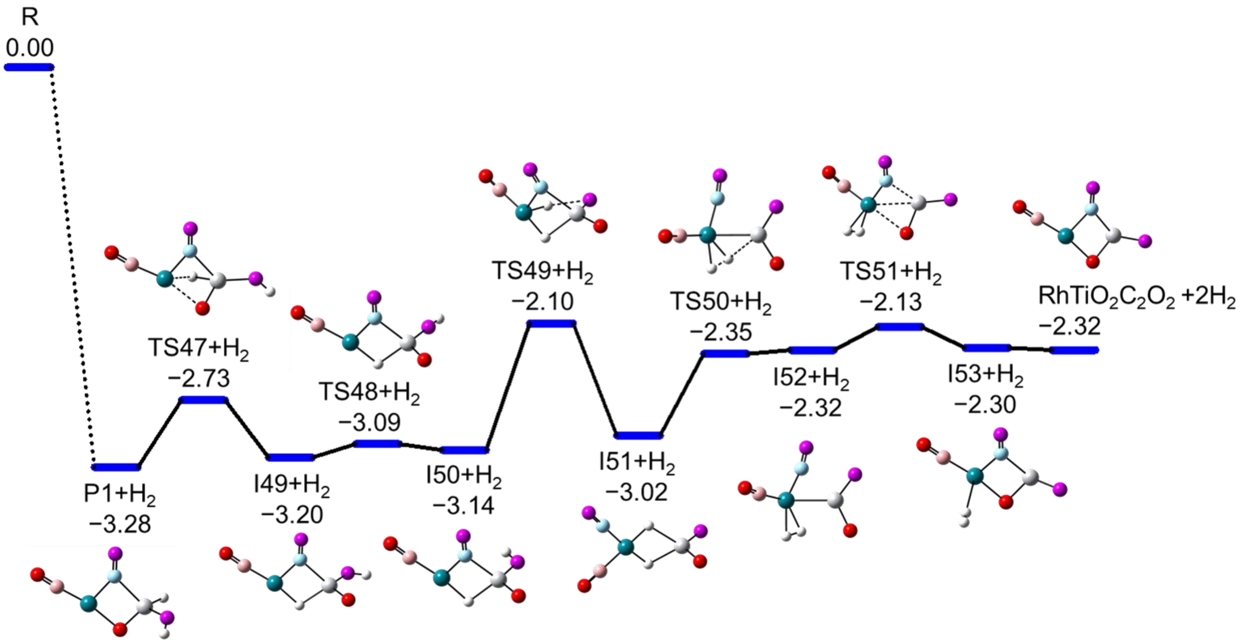

Figure S12. DFT calculated potential energy profile for $RhTiO_2CH_2CO_2^-$ (P1) + H_2 → $RhTiO_2C_2O_2^-$ + $2H_2$. The zero−point vibration−corrected energies (ΔH_0, in unit of eV) of the reaction intermediates, transition states, and products with respect to the separated reactants ($RhTiO_2^-$ + CH_4 + CO_2) are given.

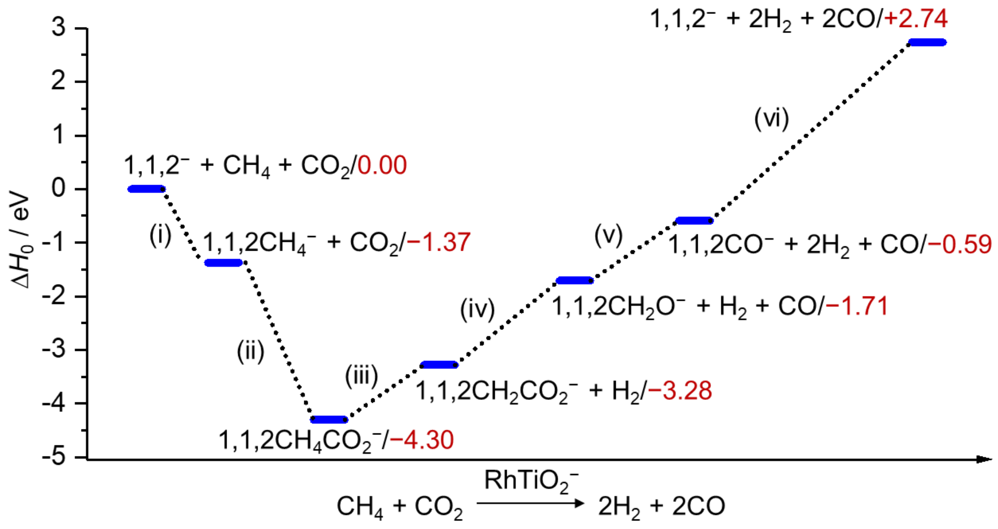

Figure S13. DFT calculated thermodynamic data for the elementary reaction of DRM to syngas catalyzed by $RhTiO_2^-$ anions. The zero−point vibration−corrected energies with respect to the separated reactants (ΔH_0) are given in eV. The $RhTiO_2^-$ and $RhTiO_2X^-$ (X= CH_4CO_2, CH_2CO_2, etc.) species are labelled as $1,1,2^-$ and $1,1,2X^-$, respectively.

Figure S13 shows the thermodynamic data, obtained from DFT calculations, for the elementary steps of DRM to syngas catalyzed by $RhTiO_2^-$. The formation of $RhTiO_2CO^-$ + $2H_2$ + CO (i−v) from the reactants is exothermic, while the final reaction step, CO desorption from $RhTiO_2CO^-$ to reform $RhTiO_2^-$ (vi), is markedly endothermic.

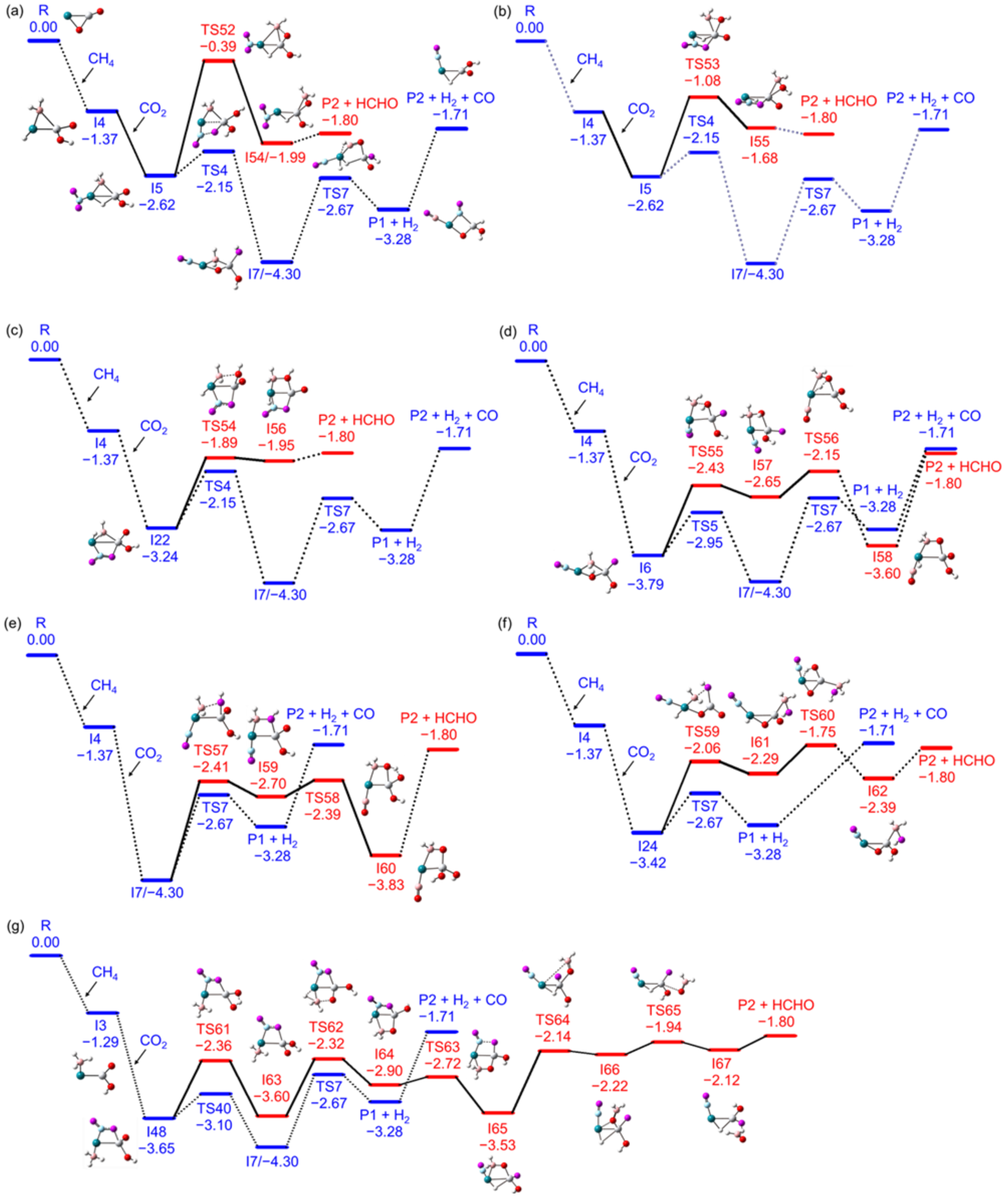

Figure S14. A comparison of the DFT calculated potential energy profiles for the formations of HCHO versus H_2 + CO from $RhTiO_2^-$ + CH_4 + CO_2. The relative energies (ΔH_0, eV) of the reaction intermediates, transition states, and products with respect to the separated reactants ($RhTiO_2^-$ + CH_4 + CO_2) are given.

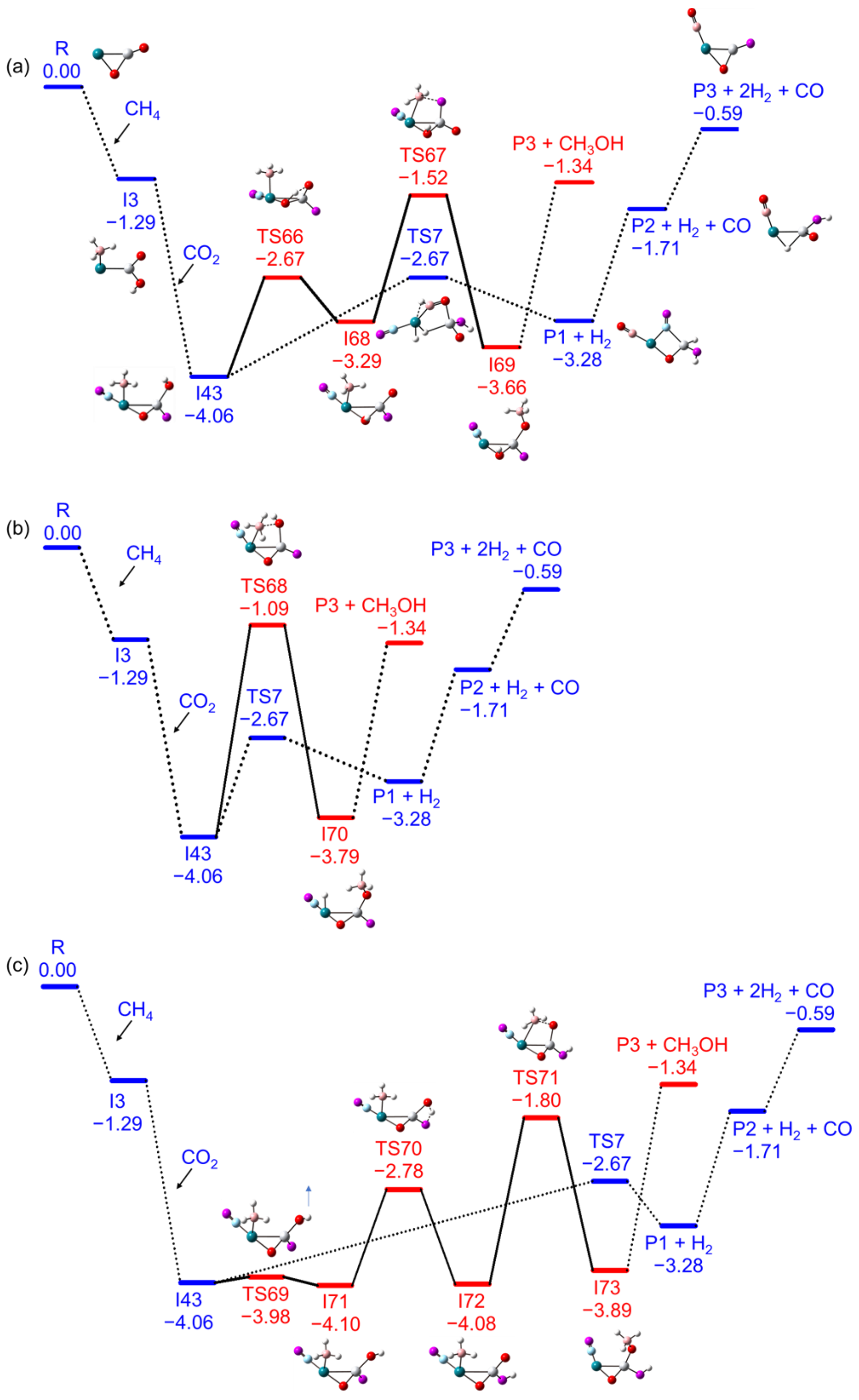

Figure S15. A comparison of the DFT calculated potential energy profiles for the formations of CH_3OH versus $2H_2 + CO$ from $RhTiO_2^- + CH_4 + CO_2$. The relative energies (ΔH_0, eV) of the reaction intermediates, transition states, and products with respect to the separated reactants ($RhTiO_2^- + CH_4 + CO_2$) are given. It is very important to note that once the reaction complex $RhTiO_2CH_4CO_2^-$ desorbs the simple molecule H_2 ($RhTiO_2CH_4CO_2^- \rightarrow RhTiO_2CH_2CO_2^- + H_2$), the remaining system $RhTiO_2CH_2CO_2^-$ will never have the chance to form CH_3OH.

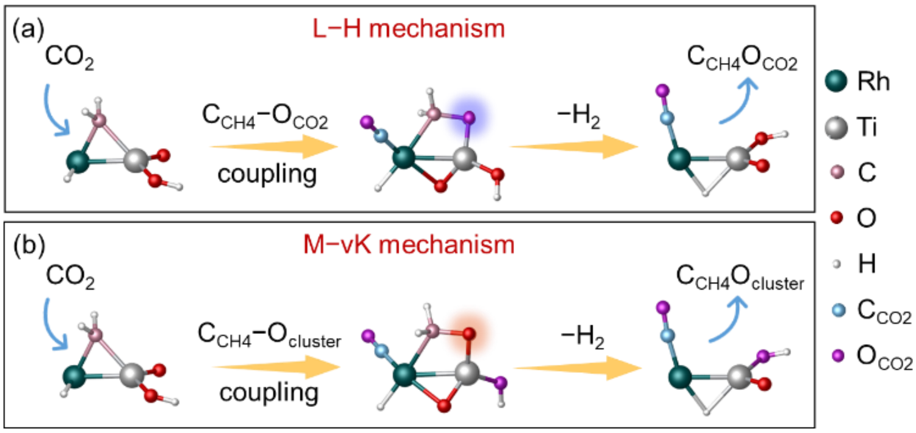

Figure S16. Two different mechanisms of DRM to syngas over $RhTiO_2^-$. Different colors for the O atom from CO_2 molecule and the $RhTiO_2^-$ cluster are shown.

The reaction mechanisms of DRM to syngas catalyzed by oxide-supported metal catalysts have attracted considerable attention. Two different proposals, either involving the Langmuir-Hinshelwood (L−H) or the Mars-van Krevelen (M−vK) mechanism, have been discussed to date.[12] Detailed mechanistic studies by isotopic labeling experiments and theoretical calculations reveal that these two mechanisms operate simultaneously in our reaction system of DRM to syngas over $RhTiO_2^-$ anions (Figure S16). After dissociative co-adsorption of CH_4 and CO_2 onto $RhTiO_2^-$, the intermediate species [CH_2] can form [CH_2O] moiety and then dehydrogenates to generate $C_{CH4}O_{CO2}$ (Figures S16a and S17).[13] Alternatively, the CH_2 fragment can be directly oxidized by the [TiO_2] cluster-support to become $C_{CH4}O_{cluster}$, the process of which corresponds to the M−vK mechanism (Figure S16b).[14]

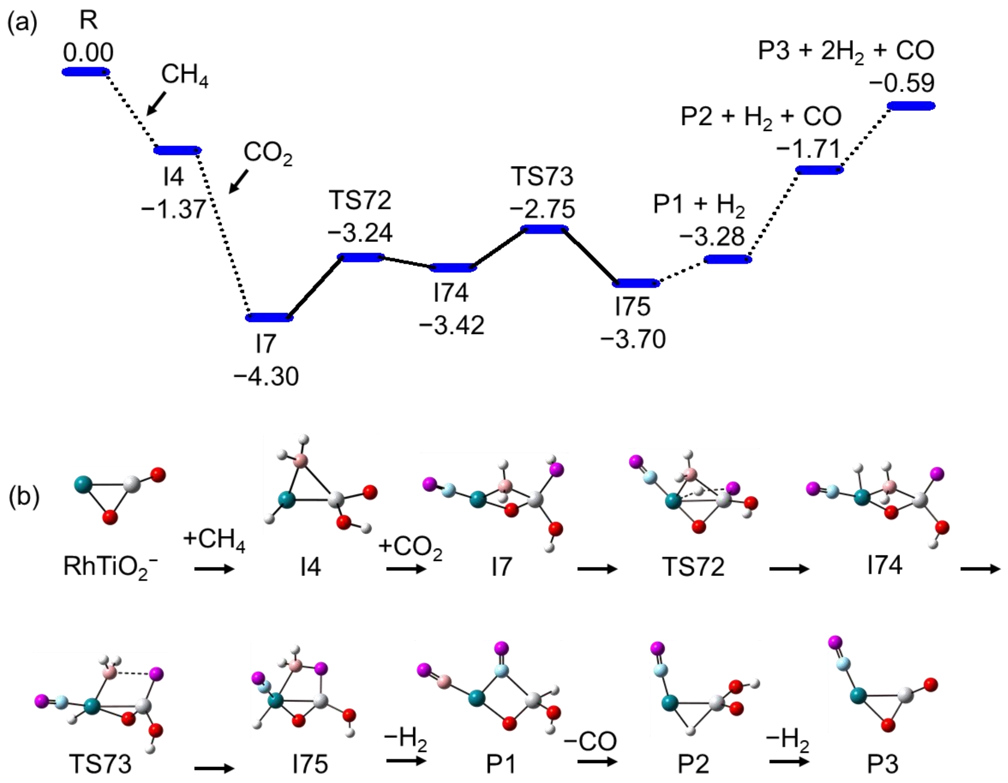

Figure S17. DFT calculated potential energy profile for the coupling of C_{CH4} and O_{CO2}. The zero−point vibration−corrected energies (ΔH_0, in unit of eV) of the reaction intermediates, transition states, and products with respect to the separated reactants ($RhTiO_2^-$ + CH_4 + CO_2) are given.

5. References

[1] Z. Yuan, Y.-X. Zhao, X.-N. Li, S.-G. He, *Int. J. Mass Spectrom.* **2013**, *354-355*, 105-112.

[2] Z. Yuan, Z.-Y. Li; Z.-X.Zhou, Q.-Y. Liu, Y.-X. Zhao, S.-G. He, *J. Phys. Chem. C* **2014,** 118 (27), 14967-14976.

[3] X.-N. Wu, B. Xu, J.-H. Meng, S.-G. He, *Int. J. Mass Spectrom.* **2012**, *310*, 57-64.

[4] Q.-Y. Liu, L. Hu, Z.-Y. Li, C.-G. Ning, J.-B. Ma, H. Chen, S.-G. He, *J. Chem. Phys.* **2015**, 164301.

[5] M. J. Frisch, G. W. Trucks, H. B. Schlegel, G. E. Scuseria, M. A. Robb, J. R. Cheeseman, G.Scalmani, V. Barone, B. Mennucci, G. A. Petersson, H. Nakatsuji, M. Caricato, X. Li, H. P. Hratchian, A. F. Izmaylov, J. Bloino, G. Zheng, J. L. Sonnenberg, M. Hada, M. Ehara, K. Toyota, R. Fukuda, J. Hasegawa, M. Ishida, T. Nakajima, Y. Honda, O. Kitao, H. Nakai, T. Vreven, J. J. A. Montgomery, J. E. Peralta, F. Ogliaro, M. Bearpark, J. J. Heyd, E. Brothers, K. N. Kudin, V. N. Staroverov, R. Kobayashi, J. Normand, K. Raghavachari, A. Rendell, J. C. Burant, S. S. Iyengar, J. Tomasi, M. Cossi, N. Rega, J. M. Millam, M. Klene, J. E. Knox, J. B. Cross, V. Bakken, C. Adamo, J. Jaramillo, R. Gomperts, R. E. Stratmann, O. Yazyev, A. J. Austin, R. Cammi, C. Pomelli, J. W. Ochterski, R. L. Martin, K. Morokuma, V. G. Zakrzewski, G. A. Voth, P. Salvador, J. J. Dannenberg, S. Dapprich, A. D. Daniels, O. Farkas, J. B. Foresman, J. V. Ortiz, J. Cioslowski, a. D. J. Fox, Gaussian 09, Revision A.01; Gaussian, Inc., Wallingford, CT, **2009**.

[6] J. Tao, J. P. Perdew, V. N. Staroverov, G. E. Scuseria, *Phys. Rev. Lett.* **2003**, *91*, 146401.

[7] Yang, Y.; Yang, B.; Zhao, Y.-X.; Jiang, L.-X.; Li, Z.-Y.; Ren, Y.; Xu, H.-G.; Zheng, W.-J.; He, S.-G. *Angew. Chem. Int. Ed.* **2019,** *58*, 17287-17292.

[8] Y. -X. Zhao, B. Yang, H. -F. Li, Y. Zhang, Y. Yang, Q. -Y. Liu, H. -G. Xu, W. -J. Zheng, S. -G. He, *Angew. Chem. Int. Ed.* **2020**, *59*, 21216-21223.

[9] A. Schafer, C. Huber, R.Ahlrichs, *J. Chem. Phys.* **1994**, *100*, 5829-5835.

[10] M. Dolg, H. Stoll, H. Preuss, *J. Chem. Phys.* **1989**, *90*, 1730-1734.

[11] I. Berente, G. Náray-Szabó, *J. Phys. Chem. A* **2006**, *110*, 772-778.

[12] K. Wittich, M. Kraemer, N. Bottke, S. A. Schunk, *Chemcatchem* **2020**, *12*, 2130-2147

[13] J, M. Wei. E. Iglesia, *J. Phys. Chem. B* **2004**, *108*, 7253-7262

[14] P. Ferreira-Aparicio, I. Rodríguez-Ramos, J. A. Anderson, A. Guerrero-Ruiz. *Appl. Catal. A: Gen.* **2000**, *202*, 183-196.
